# The Effect of Leveling the Curve of Spee on the Inclination of Mandibular Incisors: A Retrospective Cephalometric Study

**DOI:** 10.3290/j.ohpd.b3666501

**Published:** 2022-12-12

**Authors:** Domino A. J. Bernini, Theodore Eliades, Raphael Patcas, Spyridon N. Papageorgiou, Vasiliki Koretsi

**Affiliations:** a Postgraduate Student, Clinic of Orthodontics and Pediatric Dentistry, Center of Dental Medicine, University of Zürich, Switzerland. Data acquisition and validation, draft preparation.; b Professor and Chair, Clinic of Orthodontics and Pediatric Dentistry, Center of Dental Medicine, University of Zürich, Switzerland. Conceived the idea, supervision, reviewed the final manuscript.; c Senior Research and Teaching Assistant, Clinic of Orthodontics and Pediatric Dentistry, Center of Dental Medicine, University of Zürich, Switzerland. Protocol, design, reviewed the final manuscript.; d Senior Research and Teaching Assistant, Clinic of Orthodontics and Pediatric Dentistry, Center of Dental Medicine, University of Zürich, Switzerland. Protocol, design, statistical analysis of the data, wrote and reviewed the final manuscript.; e Senior Research and Teaching Assistant, Clinic of Orthodontics and Pediatric Dentistry, Center of Dental Medicine, University of Zürich, Switzerland. Protocol, design, administration, data acquisition and validation, wrote and reviewed the final manuscript.

**Keywords:** cephalometry, Curve of Spee, fixed appliances, mandibular incisors, proclination

## Abstract

**Purpose:**

To assess mandibular incisor inclination after leveling the curve of Spee (CoS) in patients treated with fixed appliances.

**Materials and Methods:**

This was a retrospective study, which included 80 consecutive patients with a mild CoS treated without extraction but with various biomechanical approaches. The depth of CoS was digitally measured on scanned plaster casts and mandibular incisor inclination was assessed with lateral cephalograms pre- and post-treatment. Patients were treated with 0.018”-slot edgewise fixed appliances and cinched back wires. Data were analyzed using linear regression modeling at 5%.

**Results:**

A total of 80 patients (40% female; mean age 13.8 years) were included with mean ANB = 4.4 ± 1.9°, mean SN/ML = 31.7 ± 4.7°, mean L1/ML = 95.0 ± 7.7°, and a mean depth of CoS = 1.1 ± 0.4 mm. The depth of CoS was leveled by -0.85 ± 0.39 mm to a post-treatment median of 0.18 mm (interquartile range = 0.09 to 0.35 mm). A small mandibular incisor proclination was observed through treatment (2.49 ± 9.1°), but this was not associated with the reduction in the depth of CoS (p > 0.05) and no statistically significant modifying effect from the different treatment mechanics was observed.

**Conclusion:**

Under the limitations of this study, leveling a mild CoS was not associated with mandibular incisor proclination during fixed-appliance treatment.

Supplementary data

**Supplementary Table 1:** Definition of cephalometric angles for the assessment of mandibular incisor inclination

Cephalometric angle	Definition
L1/ML	The angle between the long axis of the most prominent mandibular incisorxxx and the mandibular plane (a line connecting Gonion to Menton)
L1/NB	The angle between the long axis of the most prominent mandibular incisor and the NB-plane, defined by Nasion and B-point
L1/NPg	The angle between the long axis of the most prominent mandibular incisor and the NPg-plane, defined by Nasion and Pogonion
L1/OP	The angle between the long axis of the most prominent mandibular incisor and the occlusal plane, defined posteriorly by the distal cusps of the first molars and anteriorly by half the overbite of incisors

**Supplementary Table 2:** Regression of incisor inclination at T_2_ on the change in depth of the Curve of Spee between T_2_–T_1_ (with incisor inclination at T_1_ as covariate)

Variable	Unit	b	95% CI	p
L1/ML	Per millimetre	0.38	-3.36, 4.12	0.84
L1/NB	Per millimetre	-0.20	-4.04, 3.65	0.92
L1/APg	Per millimetre	0.10	-3.43, 3.63	0.96
L1/OP	Per millimetre	-0.44	-3.86, 2.99	0.80

b: unstandardised regression coefficient; CI, confidence interval.

**Supplementary Table 3:** Regression of incisor inclination at T_2_ (with incisor inclination at T_1_ as covariate) on various potential confounders

Variable	Age	Sex	Overjet	Overbite	Lower space	Molar relationship	ANB	SNA	SNB	ML/NL	SN/ML
L1/ML	0.71	**0.16**	0.70	**0.04**	**0.14**	0.95	0.44	0.71	0.37	**0.18**	**0.03**
L1/NB	0.39	**0.04**	**0.10**	**<0.001**	**0.04**	0.39	**0.003**	0.25	0.61	**0.02**	**0.11**
L1/APg	0.49	0.59	**0.09**	**<0.001**	**0.68**	0.63	0.57	0.73	0.98	**0.10**	0.62
L1/OP	0.91	0.35	**0.03**	**0.001**	**0.09**	0.78	**0.04**	0.79	0.35	**0.12**	0.58

Only p-values are reported. Confounders with at least one statistically significant effect on any inclination variable (in bold) were selected to be checked for covariate adjustment (Supplementary Table 4).

**Supplementary Table 4:** Selection of covariates to be entered in adjusted models, using the change-in-estimate method with a threshold of 10% (in bold)

Variable	L1/ML		L1/NB		L1/APg		L1/OP	
	b for Spee	Change %	b for Spee	Change %	b for Spee	Change %	b for Spee	Change %
Crude	0.1822	Reference	0.0350	Reference	-0.0582	Reference	0.4791	Reference
Sex	**-** **0.1437**	**>100%**	**-** **0.4621**	**>100%**	**-** **0.1758**	**>100%**	**0.6950**	**45%**
Overjet	**0.0470**	**74%**	**-** **0.6027**	**>100%**	**-** **0.6702**	**>100%**	**1.2446**	**>100%**
Overbite	**0.6454**	**>100%**	**0.8184**	**>100%**	**0.8335**	**>100%**	**-** **0.1733**	**>100%**
Lower space	**-** **0.0190**	**>100%**	**-** **0.2520**	**>100%**	**-** **0.0146**	**75%**	**0.7021**	**47%**
ANB	**0.1238**	**32%**	**-** **0.2379**	**>100%**	**–**	**24%**	**0.6195**	**29%**
ML/NL	**0.1512**	**17%**	**–**	**>100%**	**-** **0.0641**	**10%**	0.5114	7%
SN/ML	**-** **0.1126**	**>100%**	**0.1590**	**>100%**	**-** **0.0094**	**84%**	0.4343	9%

b: unstandardised regression coefficient.

**Supplementary Table 5:** Effect of the depth of the Curve of Spee at T_1_ on incisor inclination at T_2_ (with incisor inclination at T_1_ as covariate) using either crude models or models adjusted for selected baseline covariates (in Supplementary Table 3)

Adjustment for	L1/ML		L1/NB		L1/APg		L1/OP	
	b (95% CI)	p	b (95% CI)	p	b (95% CI)	p	b (95% CI)	p
Only T _1_ incisor inclination (crude)	0.18 (-3.39, 3.75)	0.92	0.04 (-3.63, 3.70)	0.99	-0.06 (-3.43, 3.31)	0.97	0.48 (-2.79, 3.74)	0.77
Sex	-0.14 (-3.72, 3.43)	0.94	-0.46 (-4.09, 3.17)	0.80	-0.18 (-3.59, 3.23)	0.92	0.70 (-2.60, 3.99)	0.68
Overjet	0.05 (-3.61, 3.71)	0.98	-0.60 (-4.31, 3.10)	0.75	-0.67 (-4.06, 2.72)	0.70	1.24 (-2.01, 4.49)	0.45
Overbite	0.65 (-2.88, 4.17)	0.72	0.82 (-2.61, 4.25)	0.64	0.83 (-2.19, 3.85)	0.58	-0.17 (-3.26, 2.91)	0.91
Lower space	-0.02 (-3.57, 3.53)	0.99	-0.25 (-3.86, 3.36)	0.89	-0.01 (-3.41, 3.38)	0.99	0.70 (-2.53, 3.94)	0.67
ANB	0.12 (-3.46, 3.71)	0.95	-0.24 (-3.73, 3.25)	0.89	-0.07 (-3.46, 3.31)	0.97	0.62 (-2.59, 3.82)	0.70
ML/NL	0.15 (-3.40, 3.70)	0.93	-0.05 (-3.61, 3.51)	0.98	-0.06 (-3.39, 3.27)	0.97	NT	NT
SN/ML	-0.11 (-3.60, 3.37)	0.95	0.16 (-3.48, 3.79)	0.93	-0.01 -3.40, 3.38)	1.00	NT	NT

b: unstandardised regression coefficient; CI: confidence interval; NT: not tested.

**Supplementary Table 6:** Evaluation of repeatability and agreement of duplicate intra- and inter-examiner measurements

Variable	Assessment	CCC (95% CI)	Difference (95% LoA)	p *
ANB	Intra-examiner	0.962 (0.940, 0.983)	0.060 (-0.949, 1.069)	0.62
SNA	Intra-examiner	0.989 (0.983, 0.995)	0.011 (-0.979, 1.002)	0.98
SNB	Intra-examiner	0.986 (0.978, 0.994)	-0.049 (-1.081, 0.983)	0.46
ML/NL	Intra-examiner	0.993 (0.990, 0.997)	0.017 (-1.064, 1.097)	0.85
SN/ML	Intra-examiner	0.993 (0.990, 0.997)	-0.107 (-1.177, 0.962)	0.08
L1/ML	Intra-examiner	0.992 (0.988, 0.996)	0.038 (-1.876, 1.951)	0.53
L1/NB	Intra-examiner	0.989 (0.983, 0.995)	-0.119 (-2.172, 1.935)	0.61
L1/APg	Intra-examiner	0.981 (0.970, 0.991)	-0.199 (-2.752, 2.355)	0.39
L1/OP	Intra-examiner	0.988 (0.981, 0.994)	-0.059 (-2.051, 1.933)	0.26
Spee	Intra-examiner	0.999 (0.998, 1.000)	0.010 (-0.033, 0.054)	0.009
ANB	Inter-examiner	0.961 (0.940, 0.982)	0.253 (-0.654, 1.160)	0.002
SNA	Inter-examiner	0.989 (0.982, 0.995)	0.163 (-0.909, 1.235)	0.12
SNB	Inter-examiner	0.986 (0.979, 0.994)	-0.090 (-1.151, 0.971)	0.47
ML/NL	Inter-examiner	0.989 (0.984, 0.995)	0.149 (-1.188, 1.486)	0.002
SN/ML	Inter-examiner	0.992 (0.988, 0.997)	0.179 (-0.989, 1.347)	0.03
L1/ML	Inter-examiner	0.996 (0.994, 0.998)	-0.070 (-0.991, 0.852)	0.53
L1/NB	Inter-examiner	0.993 (0.989, 0.997)	0.019 (-1.188, 1.225)	0.80
L1/APg	Inter-examiner	0.985 (0.977, 0.993)	-0.389 (-1.848, 1.069)	0.001
L1/OP	Inter-examiner	0.975 (0.962, 0.989)	0.025 (-1.843, 1.894)	0.93
Spee	Inter-examiner	0.999 (0.999, 1.000)	0.001 (-0.033, 0.036)	0.83

*From Bradley-Blackwood F-test. CCC: concordance correlation coefficient; CI: confidence interval; LoA: limits of agreement.

The curve of Spee (CoS) naturally characterises the mammalian dentition and was first described for the human dentition in 1890.^18,24^ The CoS is less excessive in the deciduous dentition, starts establishing itself with the eruption of mandibular first molars and permanent incisors,^15^ and remains more or less stable during adulthood.^10^ Initially, the functional role of CoS was described as maximising tooth contacts during chewing,^24^ yet other theories have also been proposed. Although there is limited scientific evidence, the CoS is thought to mitigate potential adversely positioned articular surfaces of the condyles, separate third molars during opening, maximise muscle force during working bite, and minimise joint reactions from bite forces.^3,19^ The concept of CoS, although it is variously approached, is clinically significant not only in orthodontics but in reconstructive dentistry as well.^15^

According to Andrews’^2^ six keys to normal occlusion, it might be prudent to therapeutically modify the plane of occlusion until it is somewhat flat or reverse to allow for best intercuspation and compensate for its post-treatment tendency to deepen. Despite not being fully scientifically justified, leveling CoS is often set as a treatment goal in contemporary orthodontics. Leveling of the CoS may be accomplished by movements of one or more tooth segments, such as uprighting of molars, extrusion of premolars, and flaring or intrusion of incisors,^23^ with the patient’s diagnostics and treatment goals dictating the optimal approach. However, there are hints that the amount of incisor flaring during leveling and alignment strongly correlates both to the baseline depth of CoS as well as baseline crowding,^12,26^ independently of the treatment mechanics used.

Because of the interplay of incisor flaring with both depth of CoS and crowding, investigations on the effect of leveling the CoS on the inclination of mandibular incisors should ideally control for initial crowding. Therefore, aim of the present study was primarily to assess the effect of leveling the CoS with fixed appliances on the inclination of mandibular incisors and secondarily to assess the potentially confounding effect of baseline crowding.

## Materials and Methods

### Design, Sample Size, Participants

This retrospective study was based on the archives of patients treated at the Clinic of Orthodontics and Pediatric Dentistry, Center of Dental Medicine, University of Zürich, Switzerland. All patients or their legal guardians signed an informed consent prior to their treatment initiation. The protocol of the study was developed in advance and was not registered.

No formal sample size calculation was performed, since this study aimed to include a convenience sample of all eligible patients treated with fixed appliances in the last decade and having available documentation. However, previous studies assessing the relationship between CoS and incisor inclination included samples of 28^1^ or 50^21^ patients, and the present study improves considerably upon them.

Case selection was performed according to the following eligibility criteria: complete diagnostic records of good quality (lateral cephalometric radiographs and dental casts), non-extraction treatment with fixed appliances for leveling the CoS, no missing teeth in the lower arch, and no tooth anomalies (position, form, and eruption) in the lower arch. Case selection did not take into account sagittal occlusal relationship, treatment mechanics, or treatment outcome.

### Interventions and Outcomes

Patients were treated by postgraduate orthodontic students under the supervision of experienced clinical instructors according to the protocol of the clinic. A 0.018”-slot edgewise fixed appliance was fitted and the archwire sequence typically included 0.014”, 0.016”, and 0.016”x0.022” nickel-titanium wires. Sometimes, 0.012” and 0.016”x0.016” nickel-titanium wires were inserted, while all wires were cinched back.

CoS leveling mechanics used in the selected sample involved a wide range of approaches including: segmented arch technique,^8^ reverse curve of Spee with continuous archwires, step-down bends, intrusive utility arches, tip-back bends on continuous stainless steel wires, and leveling with continuous archwires.

After patient selection, diagnostic records at T_1_ (before bonding) and T_2_ (after debonding) were obtained and digitised. Lateral cephalometric radiographs and plaster casts corresponding to the aforementioned timepoints were scanned (Perfection V750 Pro, SEIKO Epson; Suwa, Japan; and E1 Orthodontic, 3Shape; Copenhagen, Denmark, respectively). Subsequent measurements were performed with OnyxCeph^3^ (Image Instruments; Chemnitz, Germany) for lateral cephalometric radiographs and with Ortho Analyzer (3Shape) for digitised dental casts.

The inclination of the mandibular incisors was assessed with the cephalometric angle L1/ML as the primary measurement and also supplemented with the angles L1/NB, L1/NPg, and L1/OP to assess differences according to the reference plane chosen (Supplementary Table 1). The depth of CoS was measured as the perpendicular distance from a reference line connecting the distobuccal cusp of mandibular first molars with a point corresponding to the average height of the four mandibular incisors. Three perpendicular distances were collected per side (from the reference line to the mesiobuccal cusp of first molars, buccal cusps of first and second premolars), then the greatest value per side was considered. Finally, the average of the greatest value of the right and left sides was calculated and analysed.

The following characteristics and potential confounders were also extracted for each patient: age, sex, overjet, overbite, molar relationship, lower arch space availability, ANB, SNB, SNA, ML/NL, SN/ML, and treatment mechanics used.

### Measurement Method Error and Statistical Analyses

A sample of 50 patients was randomly chosen and measured by two of the authors, while another random sample of 50 patients was remeasured by the first author after 2 weeks. Repeatability and agreement of the measurements were assessed with the concordance correlation coefficient^14^ and the Bland-Altman method,^6^ respectively.

Normality was checked through visual graph inspection and formally with the Shapiro-Wilk test. Descriptive statistics were calculated including absolute/relative frequencies and means with standard deviations (SDs) for normal data or medians with interquartile ranges (IQRs) for non-normal data. Differences before and after treatment were assessed with paired Student’s t-tests or Wilcoxon signed-rank tests, accordingly. Linear regression modeling was used to assess the effect of the depth of CoS at T_1_ on incisor inclination at T_2_ (with incisor inclination at T_1_ as a covariate) and reported as the unstandardised regression coefficient (b) and its 95% confidence intervals (CIs). Adjusted analyses were conducted using the change-in-estimate method to select potential confounders with a minimum of 10% change set as the cut-off^13^ for variables with at least one statistically significant effect on any inclination variable. Alpha was set at a two-sided p < 0.05, all analyses were performed in Stata SE 14.2 (StataCorp; College Station, TX, USA), and the data set was openly provided.^4^

## Results

Eighty patients, whose mean age was 13.8 years (SD 3.7 years) and from whom 60% (n = 48) were males, were included in this study (Table 1). Half of them (46%, n = 37) had a Class-II molar relationship, and the sample presented a mean overjet of 4.3 ± 1.8 mm, mean overbite of 5.4 ± 1.2 mm, and mean lower arch space of 0.3 ± 2.4 mm. On average, a skeletal Class-I pattern was seen (ANB = 4.4 ± 1.9°), with increased mandibular divergence (SN/ML = 31.7 ± 4.7°) and neutral mandibular incisor inclination (L1/ML = 95 ± 7.7°).

**Table 1:** Characteristics of the included sample (n = 80) at T_1_

Variable	Measure	Value
Age (years)	Mean (SD)	13.8 (3.7)
Sex	Female – n (%)	32 (40%)
	Male – n (%)	48 (60%)
Molar relationship	I – n (%)	43 (54%)
	II – n (%)	37 (46%)
Overjet (mm)	Mean (SD)	4.3 (1.8)
Overbite (mm)	Mean (SD)	5.4 (1.2)
Lower space (mm)	Mean (SD)	0.3 (2.4)
ANB (°)	Mean (SD)	4.4 (1.9)
SNA (°)	Mean (SD)	80.9 (3.4)
SNB (°)	Mean (SD)	76.5 (3.0)
ML/NL (°)	Mean (SD)	25.7 (5.0)
SN/ML (°)	Mean (SD)	31.7 (4.7)
L1/ML (°)	Mean (SD)	95.0 (7.7)
L1/NB (°)	Mean (SD)	23.3 (7.4)
L1/APg (°)	Mean (SD)	20.7 (7.3)
L1/OP (°)	Median (IQR)	70.9 (67.6, 75.7)
Spee (mm)	Mean (SD)	1.1 (0.4)

IQR: interquartile range; SD: standard deviation.

Considerable changes due to treatment were observed in the CoS, which was reduced by 77.9% to a post-treatment median depth of 0.2 mm (IQR = 0.1 to 0.4 mm) (Table 2). Additionally, a small proclination of the mandibular incisors was noticed for L1/ML (+2.5°); this was independent of the reference plane used (+2.4° for L1/NB, +3.8° for L1/APg, and -2.4° for L1/OP). However, no statistically significant association was detected between mandibular incisor inclination and depth of CoS for all measurements (Table 3). Post-hoc analysis assessing the effect of the amount of CoS leveling (T_2_–T_1_ change in depth) on mandibular incisor inclination also yielded similar results (Supplementary Table 2).

**Table 2:** Changes in incisor inclination and depth of the Curve of Spee throughout treatment (T_2_–T_1_)

Variable	T_1_*	T_2_*	Change	Change %	p
L1/ML (°)	95.0 (7.7)	97.5 (6.5)	+2.5	+3.2	0.02^†^
L1/NB (°)	23.3 (7.4)	25.7 (6.7)	+2.4	+28.6	0.02^†^
L1/APg (°)	20.7 (7.3)	24.5 (6.2)	+3.8	+49.6	0.002^†^
L1/OP (°)	[70.9] {67.6, 75.7}	69.8 (5.9)	-2.4	-2.5	0.07^‡^
Spee (mm)	1.1 (0.4)	[0.2] {0.1, 0.4}	-0.8	-77.9	<0.001^‡^

*According to its distribution, given either as mean (standard deviation) or as [median] {interquartile range}. ^†^paired t-test; ^‡^Wilcoxon signed-rank test.

**Table 3:** Regression of incisor inclination at T_2_ on the depth of the Curve of Spee at T_1_ (with incisor inclination at T_1_ as covariate)

Variable	Unit	b	95% CI	p
L1/ML	Per mm	0.18	-3.39, 3.75	0.92
L1/NB	Per mm	0.04	-3.63, 3.70	0.99
L1/APg	Per mm	-0.06	-3.43, 3.31	0.97
L1/OP	Per mm	0.48	-2.79, 3.74	0.77

b: unstandardised regression coefficient; CI: confidence interval.

No statistically significant confounding effects were observed due to baseline characteristics, as adjusting for multiple potential confounders selected (Supplementary Tables 3 and 4) did not alter the results (Supplementary Table 5). Finally, no statistically significant modifying effect was identified on this relationship according to the different treatment mechanics used (Table 4).

**Table 4:** Effect of the depth of the Curve of Spee at T_1_ on incisor inclination at T_2_ (with incisor inclination at T_1_ as covariate) using either crude models or models adjusted for treatment mechanics

Adjustment for	Category	L1/ML		L1/NB		L1/APg		L1/OP	
		b (95% CI)	p	b (95% CI)	p	b (95% CI)	p	b (95% CI)	p
Only T_1_ incisor inclination (crude)	–	0.18 (-3.39, 3.75)	0.92	0.04 (-3.63, 3.70)	0.99	-0.06 (-3.43, 3.31)	0.97	0.48 (-2.79, 3.74)	0.77
Treatment mechanics	Segmented arches	Reference	0.78†	Reference	0.65†	Reference	0.21†	Reference	0.42
	Reverse curve of Spee	-0.67 (-6.86, 5.52)		1.68 (-4.60, 7.96)		1.92 (-3.71, 7.55)		-0.83 (-6.37, 4.72)	
	Step-down bends	-0.04 (-6.26, 6.18)		0.49 (-5.85, 6.84)		-1.28 (-6.99, 4.43)		-1.54 (-7.16, 4.07)	
	Leveling / aligning	-3.91 (-12.20, 4.38)		-4.05 (-12.55, 4.44)		-5.73 (-13.30, 1.83)		3.52 (-3.92, 10.96)	
	Intrusive utility arches	-7.41 (-21.95, 7.13)		1.89 (-12.79, 16.57)		3.67 (-9.36, 16.70)		0.91 (-12.03, 13.86)	
	Tip-back bends	-0.57 (-7.30, 6.15)		2.84 (-3.88, 9.56)		2.12 (-3.91, 8.15)		-3.50 (-9.44, 2.44)	
	Combination	-2.34 (-8.44, 3.76)		0.34 (-5.81, 6.48)		-0.22 (-5.75, 5.30)		-0.04 (-5.49, 5.40)	

b: unstandardised regression coefficient; CI: confidence interval. † overall Wald test.

Assessment for the intra- and inter-examiner reliability indicated excellent agreement (Supplementary Table 6). For all outcomes, intra- and inter-examiner concordance ranged from 0.962 to 0.999 and 0.961 to 0.999, respectively. Furthermore, Bland-Altman limits were very narrow in all instances, not exceeding 0.05 mm for CoS measurements and 1.93° for L1/ML measurements.

## Discussion

This retrospective study assessed the effect of leveling a mild CoS with fixed appliances on the inclination of mandibular incisors, revealing a lack of a consistent relationship. In contrast, Pandis et al^21^ reported an increase of 4° for each millimetre of leveling the CoS. This discrepancy between findings might be attributed to different treatment protocols as well as initial arch length discrepancy (crowding), which was 2.5 to 5.5 mm for half the patients and more than 5.5 mm for the other half of the patients in the study by Pandis et al.^21^ In contrast, the present study included patients with mostly minimal arch length discrepancy, with only 10% of the patients having crowding greater than 2.5 mm. However, previous studies demonstrated that mechanics for leveling the CoS could be more relevant than space requirements for the protrusion of mandibular incisors.^7^ Some patients in the present study were treated with the segmented arch technique,^8^ which is considered superior to continuous arches in preventing flaring of incisors.^9,25^

Moreover, the present study included mean depths of CoS of 1.1 mm, ranging from 0.26 to 2.47 mm and measured from the mandibular first molars to the mandibular incisors. Additionally, it resulted in considerable flattening of the CoS by 0.85 mm, so that the post-treatment CoS depths ranged from 0 to 0.80 mm. The depth of CoS corresponding to the developmental stage of erupted mandibular first molars and incisors reaches a mean maximum of 1.32 mm,^15^ which further increases after eruption of the mandibular second molars.^15^ The fact that CoS was not fully flattened is a common finding in the literature^5,21,22^ and might be attributed to either cusp abrasion or the fact that just the greatest measurement of the two sides was considered.

Furthermore, CoS can be leveled by many means, including uprighting molars, extruding premolars, and/or proclining or intruding incisors.^23^ It seems that the response to leveling the CoS with continuous archwires varies according to growth pattern, i.e. brachycephalic patients respond predominantly with mandibular incisor proclination and intrusion, whereas dolichocephalic patients respond mostly with extrusion and uprighting of posterior teeth.^22^ Also, statistically significantly greater reductions in interincisal angle because of incisor proclination have been reported for neutral and horizontal growth patterns compared to patients with a vertical growth pattern during leveling of the CoS with continuous archwires.^22^ In the present study, patients with several extents of mandibular divergence were analysed, including hypodivergence (n = 11 with SN/ML<27), normo-divergence (n = 32 with SN/ML between 27° and 32°), or hyperdivergence (n = 37 with SN/ML>32°), and various intrusive mechanics were used. Apart from continuous archwires with or without reverse curves, segmented arches, step-down bends, intrusive utility arches, and tip-back bends were used. No statistically significant effect on the mandibular incisor inclination after leveling the CoS was identified, and the aforementioned treatment mechanics did not ^statistically^ significantly modify that effect. The fact that no consistent relationship between CoS depths and mandibular incisor inclination was established might be attributed to the mild depths of CoS included and the fact that all wires were cinched back. Cinched back wires might minimise mesial movement of anterior teeth and better preserve initial incisor inclination.^16^ In studies reporting on increased mandibular incisor inclination after leveling CoS, wires were not cinched back,^1^ or it was not reported whether wires were cinched back.^11,20-22^

A potential limitation of the present study might be not distinguishing between an angulated and a stepped CoS. A stepped CoS would not require space for its leveling, yet an angulated CoS would increase arch length, which could be achieved by proclining incisors.^17^ Also, molar uprighting or extrusion contributing to CoS leveling were not quantified in the present study, since the focus was on the anterior teeth. With respect to the applicability of the present findings, the present study did not include patients with excessive depths of CoS and, therefore, the present findings could be relevant only in cases with a mild depth of CoS. Finally, strengths of the present study might be related to the definition of the study protocol in advance and the thorough assessment of treatment mechanics and further covariates at the statistical level, since this was a retrospective study. Moreover, the availability of the data set online may further add to the overall transparency of the study.

## Conclusion

In patients with mild depths of CoS treated with fixed orthodontic appliances without extractions, no consistent association was identified between leveling the CoS and mandibular incisor proclination.
